# Pool boiling of nanoparticle-modified surface with interlaced wettability

**DOI:** 10.1186/1556-276X-7-259

**Published:** 2012-05-18

**Authors:** Chin-Chi Hsu, Tsung-Wen Su, Ping-Hei Chen

**Affiliations:** 1 Department of Mechanical Engineering, National Taiwan University, No. 1, Sec. 4, Roosevelt Rd, Taipei, 10617, Taiwan

**Keywords:** Hydrophilic, Hydrophobic, Critical heat flux, Boiling heat transfer

## Abstract

This study investigated the pool boiling heat transfer under heating surfaces with various interlaced wettability. Nano-silica particles were used as the coating element to vary the interlaced wettability of the surface. The experimental results revealed that when the wettability of a surface is uniform, the critical heat flux increases with the more wettable surface; however, when the wettability of a surface is modified interlacedly, regardless of whether the modified region becomes more hydrophilic or hydrophobic, the critical heat flux is consistently higher than that of the isotropic surface. In addition, this study observed that critical heat flux was higher when the contact angle difference between the plain surface and the modified region was smaller.

## Background

Regarding the Nukiyama curve [1] in the heat and mass transfer textbook, the nucleate boiling region causes a higher rate of heat transfer in the boiling system. However, the nucleate boiling system is limited by the critical heat flux (CHF); therefore, CHF might be set as an index for nucleate boiling research. Several factors affect this situation, and a number of early studies reported that surface wettability is a crucial factor that affects the boiling heat transfer [2,3]. The contact angle (CA) has a substantial influence on transition boiling, and the hydrophilic surface increases the CHF and heat transfer coefficient (HTC) in boiling heat transfer [4]. Dhir and Liaw [5,6] reported that the theoretical prediction of CHF efficiently compares with the experimental data in the hydrophilic region. Thin-film coatings with alumina [7,8], zirconia [8], or silica [8,9] nanoparticles applied to modify surface wettability demonstrated considerable enhancement in the pool boiling CHF. An approximate enhancement of the CHF was observed for pool boiling in a surface modified with micro/nanoscale of zircaloy-4 [10]. Chen et al. [11] reported that a superhydrophilic surface that is created by the nanowire arrays on Si and Cu substrates can be utilized to increase the CHF by more than 100%. Kim et al. [12] reported that CHF enhancement in nanofluids is due to surface deposition of nanoparticles during boiling, and the resulting surface had significantly increased wettability. Therefore, the surface wettability is a crucial subject of boiling heat transfer. The interlaced wettability surface effect on pool boiling heat transfer was studied by Betz et al. [13], in which the interlaced wettability of a silicon surface formed with hydrophilic (CA = 7°) and hydrophobic (CA = 110°) regions. The ratio of the hydrophilic patterned area was 77.4%, and the measured CHF increased by 65% higher than that of a plain silicon surface treated with hydrofluoric acid (CA = 7°).

## Main text

### Nanoparticle coating

The purpose of this research was to alter the difference of interlaced wettability effects on pool boiling heat transfer in the same area of condition. The application of the nano-silica particle (40 nm) coating method established a variety in the various levels of wettability and introduced the interlaced wettability surface of plain-hydrophilic region and plain-hydrophobic region effects in boiling heat transfer. Various levels of wettability surfaces were formed by the silica-based coating method. A common sol–gel method was used to prepare nano-silica particles. First, the precursor was prepared by mixing solution A with solution B (1.5:100 in *v*/*v*). Solution A was composed of tetraethoxysilane and deionized (D.I.) water. The molar ratio was 1:4. Solution B was composed of ethanol and D.I. water. The molar ratio was 1:3. Finally, 4 g of nanoparticles was mixed with the precursor solution and magnetically stirred at room temperature. Step one included a coating method, which consists of airbrushing the copper block, the area of which was 1.5 × 1.5 cm^2^. The weight of the copper block was measured before coating. All of the coating experiments were conducted by airbrushing the surface at various times. The weight of the copper block was measured again after coating. We subsequently discovered the number of grams of precursor that was coated with particles. At this step, the wettability of the modified surfaces was hydrophilic. Surface roughness was measured to show the surface geometrical details. Average roughness (*R*_a_) was measured by a probe-type surface roughness analyzer (ET-4000A, KOSAKA, Chiyoda-ku, Tokyo, Japan). The *R*_a_ of the plain copper (nanoparticles uncoated) regions was approximately 0.15 μm. The weight of nanoparticles coated was 0.003 g, and *R*_a_ was 0.34 μm. Coating with more particles ensures a rougher and more wettable surface. All the details of surface roughness are showed in Table 1.

**Table 1:** The effect of CA and details of coating methods

**Regions of interlaced surface**		**Hydrophilic**		**Plain**	**Hydrophobic**	
Step 1: precursor + nanoparticle coatings (g)	0.003	0.0025	0.0008	0	0.0008	0.002
Step 2: hydrophobic material treatment	No	No	No	No	Yes	Yes
*R*_a_ (μm)	0.34	0.30	0.19	0.15	0.19	0.27
CA (°)	<10	16	55	93 to 111	123	145

*R*_a_, average roughness; CA, contact angle.

**Figure 1:**
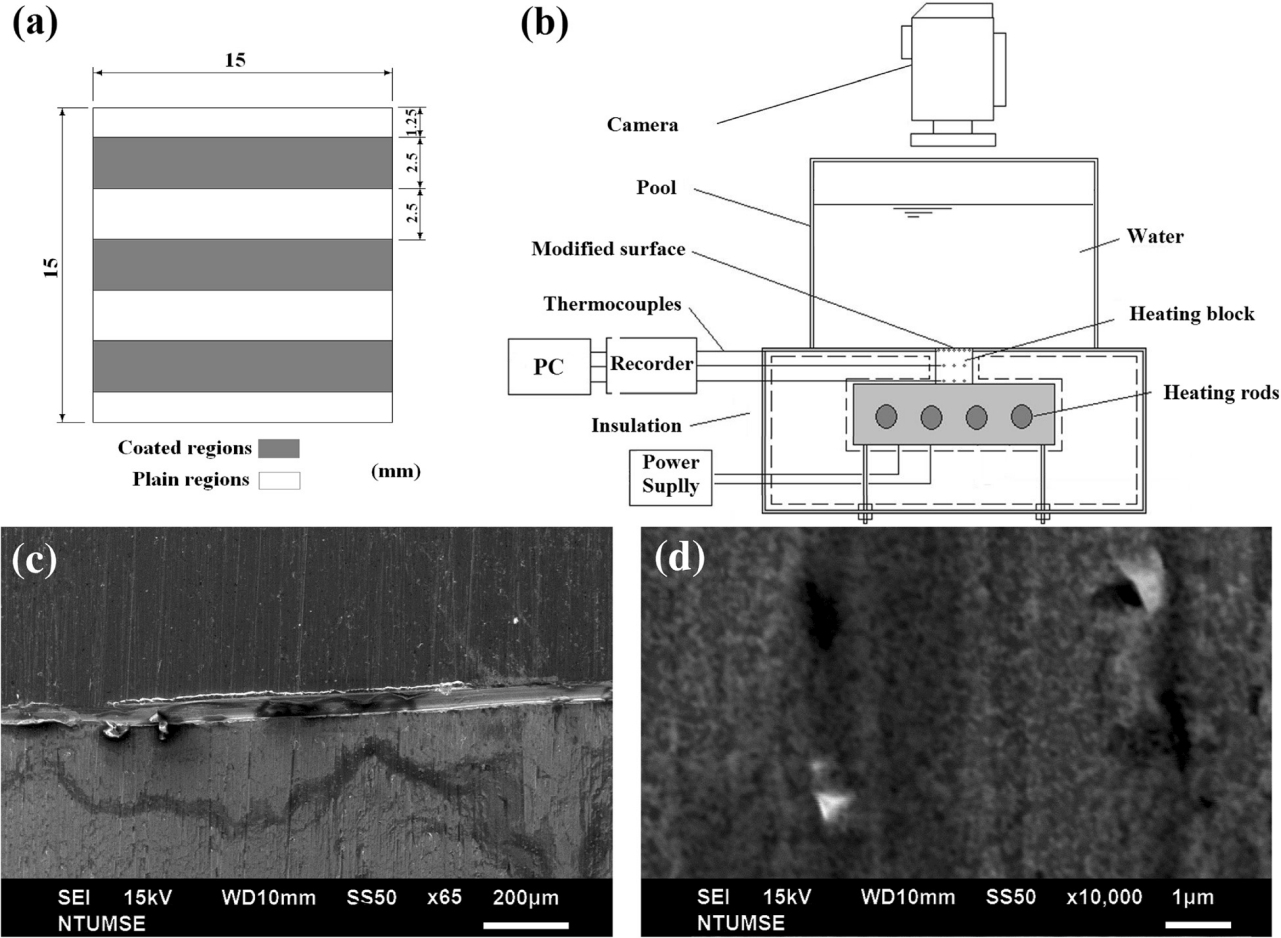
**Heating surface, experimental facility setup, and scanning electron microscopy (SEM) images.** (**a**) Heating surface with coated and uncoated (plain) areas. (**b**) Experimental facility setup of the thermal system. (**c**) SEM image of nanoparticle-coated (downside, CA = 55°) and uncoated (plain) areas (upside, CA = 105°) at a scale bar of 200 μm. (**d**) SEM image of nanoparticles coated areas at a scale bar of, 1 μm.

The second step included the preparation of hydrophobic materials by fluoro-containing mixtures. Trichloro (1H, 1H, 2H, 2H-perfluorooctyl)silane (PFOCTS) was mixed with methyl alcohol (1:100 in *v*/*v*). Subsequently, the surface wettability transitioned into a de-wetting process, in which more particles increased the surface hydrophobicity after the PFOCTS were coated. The interlaced wettability coating of this experiment fabricated a periodic and equally spaced (width is 2.5 mm and length is 15 mm) surface that consisted of both coated and uncoated regions, as shown in Figure 1a.

A type of high-density anti-coating tape was used to achieve this. The sample with a plain surface was first covered with the striped tape. All stripes were aligned with the locations of thermal couples to ensure that the readouts were accurate. The details of the procedure are followed by a series of abovementioned coating steps. The desired pattern of the surface can be modified with other wettability with nanoparticles coated. SEM image of interlaced surface is shown in Figure 1c. There is an interface between nanoparticle-coated and uncoated regions. SEM image of nanoparticle-coated region is shown in Figure 1c downside, (CA = 55°), and that of uncoated (plain) areas is shown in Figure 1c upside, (CA = 105°) at a scale bar of 200 μm. Nano-silica particles coated on copper surface can be observed in Figure 1d.

All CAs are measured before the tests, using Sindatek Model 100SB (Taipei, Taiwan) to produce schematic drawings of the CA meter. A drop volume of 2 μl was chosen for the measurements, and each region was examined using more than five values. The CAs of the plain copper surfaces after baking were approximately 93° to 111°. Interlaced wettability surfaces show two CAs in different regions. The first value is the contact angle in plain regions, and the second value is the contact angle in coated regions. CAs of samples with plain and hydrophilic regions are 105° and 55°, 105° and 33°, or 97° and 10°. Besides, CAs of samples with plain and hydrophobic regions are 111° and 142° or 93° and 123°.

### Thermal system

The heat system setup is displayed in Figure 1b. A copper block with dimensions of 1.5 cm × 1.5 cm was prepared. Holes with a depth of 0.7 mm were drilled on the sides of the copper block. The accuracy of the T-type thermocouples used in the present experiments was ±0.1°C. A thermocouple was placed in each of these holes with sink grease to reduce the contact resistance, the positions of which were at the top, side, and bottom. Each hole was separated by a space of 7 mm. The top of the copper block was the heated surface with varied surface wettability after modification. The side of the heated block was full of glass fiber for heat insulation. A glass tank with dimensions of 10 cm × 10 cm × 25 cm was placed on the top of the heated block. The opening on the top of the tank maintained at atmospheric pressure, and the pool temperature was naturally adjusted to the saturated temperature (100°C). The camera was placed on the top side of the tank to record the photos of growth bubbles. The heat source was placed under the heated block. The heating source was supplied by four 200-W electric heating rods. The control heating source passed through the electricity supplier and outputs. After heating the surface, the thermometer recorder (MX-100, Yokogawa, Musashino-shi, Tokyo, Japan) read the data that were measured by the thermocouples and transferred it to the computer for analysis. At the top position of the temperature data is the wall temperature; the temperature difference was obtained from the bottom position and the top position of the temperature data. Once the distance difference (14 mm) and copper thermal conductivity were determined, the Fourier law (Equation [1]) was used to calculate the heat flux into the boiling liquid. Through energy balance equation, the heat loss can be calculated. The percentage of heat loss through insulation was 0.77%. Uncertainties of the experimental measurement of heat flux and wall superheat were estimated using Equations [2] and [3]. The parameter U_q_ in these equations represents the uncertainty of the heat flux q and the parameter U_Tw_ represents the uncertainty of the wall temperature T_w_. A homogeneous temperature profile on the surface was obtained in this study. The accuracy of the T-type thermocouples used in the present experiments was ±0.1°C. All the thermocouples were calibrated using an OMEGA-HH41 thermistor (Stamford, CT, USA).

The maximum fabrication error of the temperature-measuring positions was 0.15 mm. The overall maximum uncertainties in the heat flux and wall temperature measurements were 5.1% and 5.8% at *q* = 100 kW/m^2^, and 1.1% and 1.6% at *q* = 1,000 kW/m^2^, respectively.

(1) $$q=K\left(\frac{T_{2}-T_{1}}{\Delta x}\right)$$

(2) $$\frac{U_{q}}{q}={\left[{\left(\frac{U_{T_{2}-T_{1}}}{T_{2}-T_{1}}\right)}^{2}+{\left(\frac{U_{\mathrm{\Delta x}}}{\Delta x}\right)}^{2}\right]}^{0.5}$$

(3) $$\frac{U_{T_{\text{w}}}}{T_{\text{w}}}={\left[{\left(\frac{U_{T_{0}-T_{\text{sat}}}}{T_{0}-T_{\text{sat}}}\right)}^{2}+{\left(\frac{U_{q}}{q}\right)}^{2}\right]}^{0.5}$$

## Discussion

**Figure 2:**
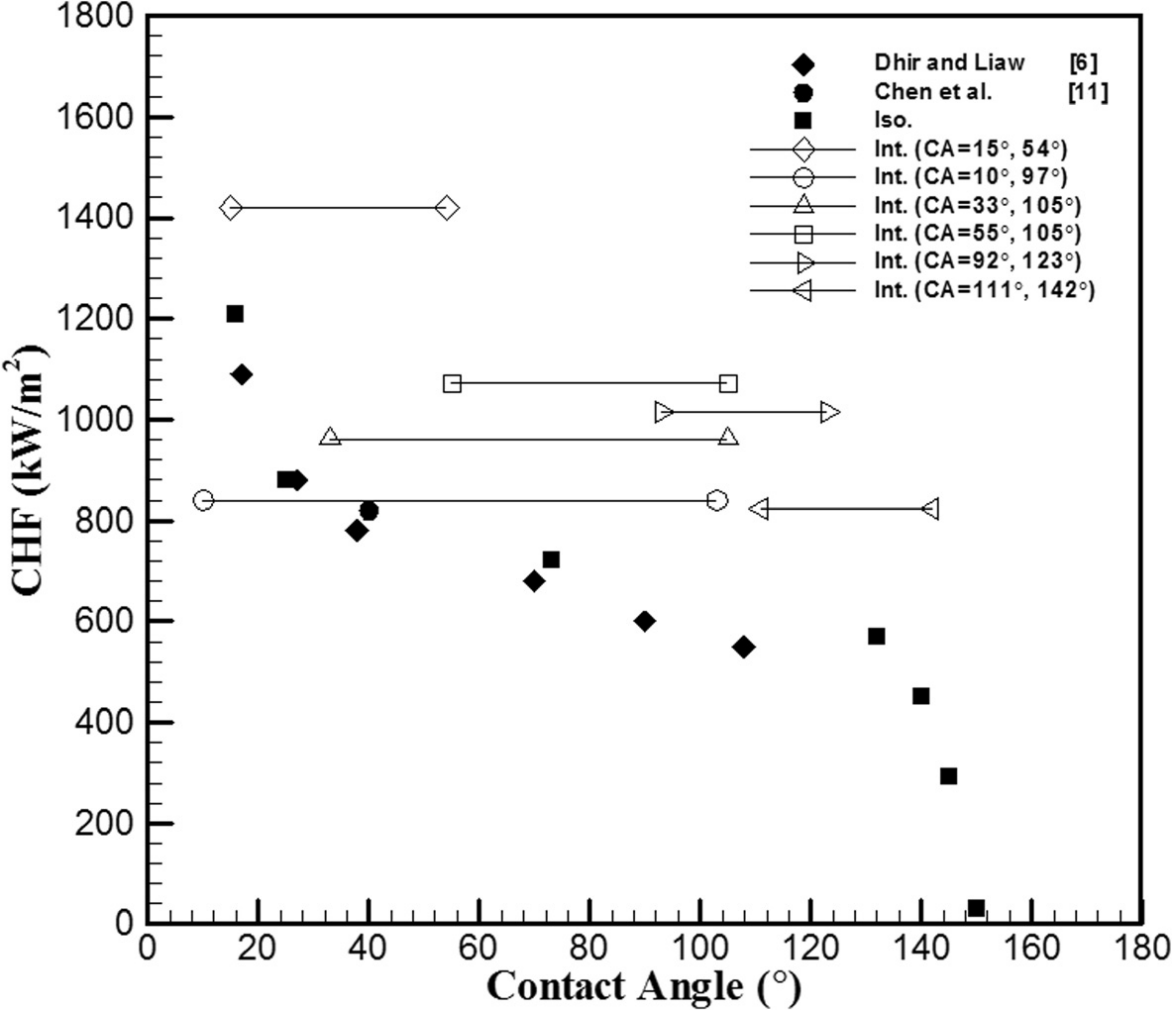
**Data with interlaced wettability are plotted against with isotropic wettability data and references data.** Iso. = isotropic wettability and Int. = interlaced wettability.

**Figure 3:**
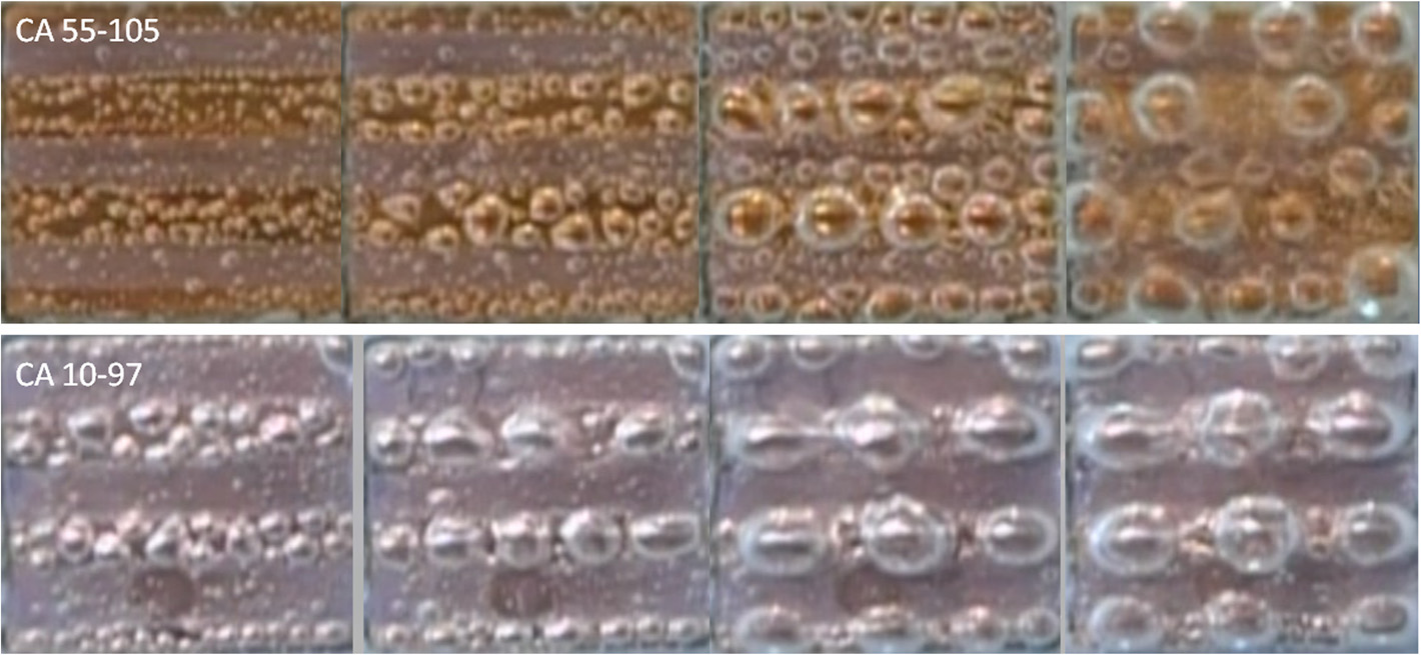
**Top view of the heating surface (interlaced wettability) during the heating process.** Wall temperatures are 85°C, 90°C, 100°C, and 110°C from the right to the left side of the image. Bubbles gathered in the lower surface (plain) regions in these cases.

**Figure 4:**
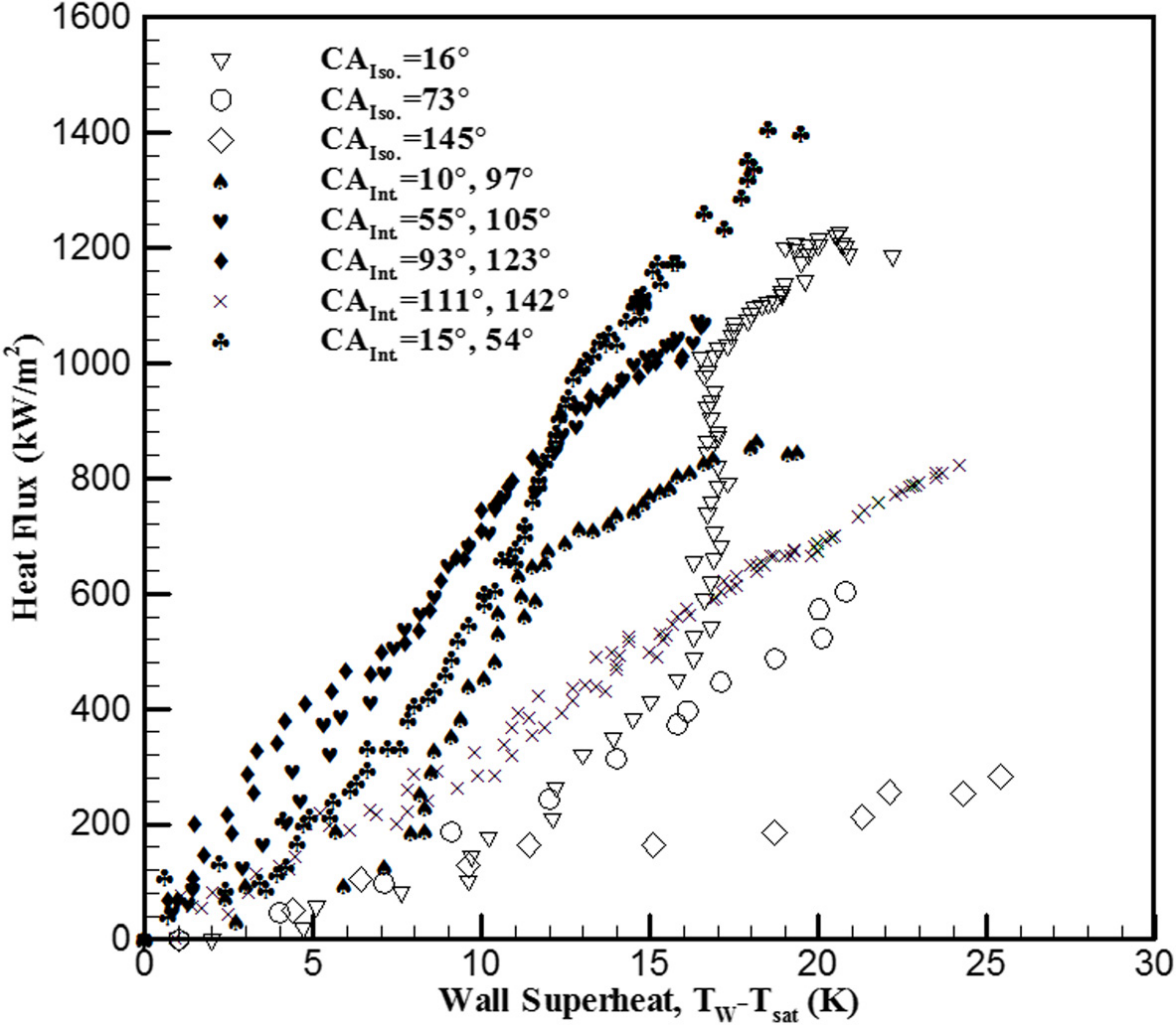
**Heat flux value plotted against wall superheat with different wettability of isotropic and interlaced surfaces.** Iso. = isotropic wettability and Int. = interlaced wettability.

**Figure 5:**
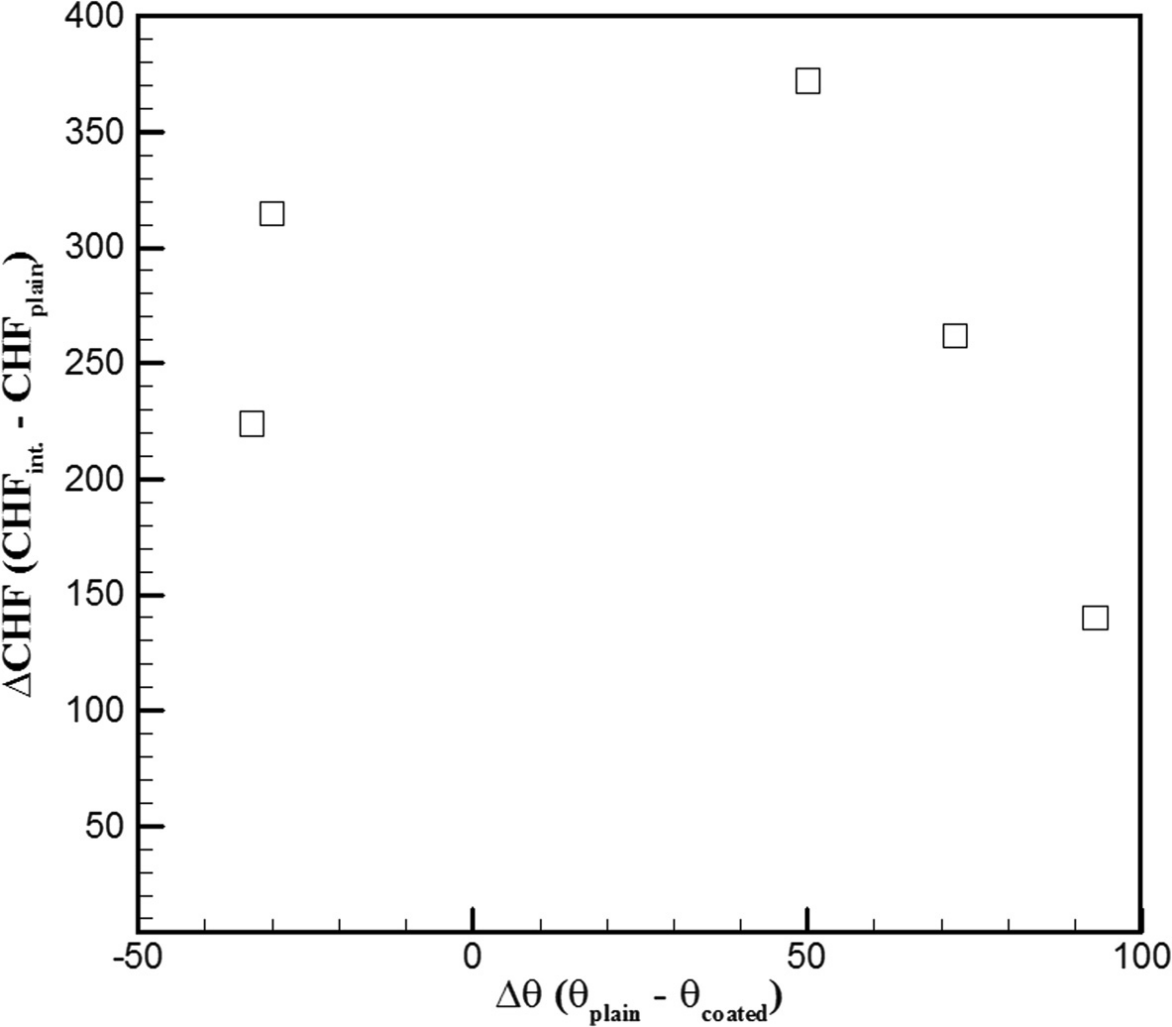
**The Δ*θ*of the interlaced surface affects the ΔCHF.**

This study reveals the main differences in the boiling heat transfer mechanism between interlaced surfaces and isotropic surfaces and provides possible explanations for the resulting value of CHF. The interlaced sample that was used in this experiment consisted of two distinct surface conditions in which slightly contaminated plain surfaces (CA = 105°) and hydrophilic surfaces (CA = 55°) were interlaced. Figure 2 shows the CHF value, which is plotted against the CA with the data of isotropic wettability, interlaced wettability, and references. As shown in Figure 2, in isotropic plain surfaces (CA = 105°), the CHF value was approximately 650 kW/m^2^, and in isotropic hydrophilic surfaces, the CHF value was approximately 810 kW/m^2^. The experiment of interlaced surfaces revealed that these two surfaces (CA = 105° and 55°) result in a higher CHF value of approximately 1,072 kW/m^2^. During the nucleate boiling regime in the heating process, as shown in Figure 3, it was observed that bubbles start nucleating on the edge plain surfaces. Some smaller bubbles that form above the hydrophilic surfaces have the tendency to migrate to the neighboring plain surfaces and combine with the existing bubbles, and the migration rate increased in conjunction with the temperature; this intensified bubble accumulation forced the unstable bubble to break off from the plain surfaces, thereby producing an upward surge of flow. This dynamic process continued throughout the nucleate boiling regime. This phenomenon helps the nucleation of bubble and increased the value of HTC by providing an efficient means of convection heat transfer. Betz et al. [13] had reported that the enhancement of HTC on patterned surfaces (hydrophobic and hydrophilic network) can be explained by the increased availability of active nucleation sites. The rate of heat transfer is mainly associated with the formations and collapse of bubbles. The heat flux value, which was plotted against the wall superheat $\left(\Delta T=T_{\text{w}}-T_{\text{sat}}\right)$ with varied wettability surfaces, is shown in Figure 4. The HTC values can be observed in the slope of Figure 4. The HTC was defined as the heat flux over wall superheat; the wall superheat is the temperature difference between the heating surface and saturation temperature of the liquid. Regarding wall superheat to heat flux, the slopes of the interlaced surfaces were greater than those of the isotropic surfaces. In other words, the data revealed that the HTC values in interlaced surfaces are superior to those in the isotropic surfaces. A higher CHF value was also obtained in another experiment with other types of interlaced surfaces (plain and near-superhydrophobic). In comparison to the isotropic surface, the near-superhydrophobic surface (CA = 145°) exhibits a lower CHF value, approximately 290 kW/m^2^ as shown in Figure 2. This signifies the formation of a vapor film between the liquid and the heated surface. However, the interlaced plain and near-superhydrophobic surfaces (CA = 111° and 142°) help nucleation and enhance CHF by preventing the early formation of a vapor film, with values of CHF ranging from 290 to 824 kW/m^2^. The areas were separated from each other, and the vapor film was more difficult to form by the near-superhydrophobic surface to sustain above the surfaces, which may block the path of heat transfer. The main differences between these two experiments (interlaced plain-hydrophilic surfaces and interlaced plain-hydrophobic surfaces) were the direction of bubble motion and the presence of bubbles. In the plain-hydrophobic case, the bubbles moved from plain surfaces to hydrophobic surfaces, and in the plain-hydrophilic case, the experiment revealed that the bubbles moved from hydrophilic surfaces to plain surfaces. In other words, bubbles are likely to move from the surfaces with lower CA to the surfaces with higher CA among the interlaced wettability surfaces. This phenomenon occurred because of the varied surface energy between two adjacent surfaces, and this surface energy difference could be an important factor of the driving force of bubble motion. The hydrophobic (or plain) surfaces were lower in surface energy, and the plain (or hydrophilic) surfaces were higher in energy. Therefore, when bubbles are formed, they move from plain (or hydrophilic) surfaces to hydrophobic (or plain) surfaces to maintain lower and more stable energy. The bubbles gathered in the lower surface regions and increased in size before departing from the heating surface. These bubble motion effects, which are due to the existence of bubbles, were no longer present, resulting in higher HTC than that in the isotropic wettability surfaces as mentioned. The results proved that combined surfaces (plain-hydrophobic or plain-hydrophilic surfaces) achieved higher CHF values. Specifically, as shown in Figure 2, the CHF of CA = 33° of the isotropic surface was approximately 850 kW/m^2^, the CHF of CA = 105° of the isotropic plain copper surface was approximately 640 kW/m^2^, and the CHF of the combined interlaced surface was approximately 962 kW/m^2^, which indicated that CHF was enhanced by approximately 50% as compared with that of the plain surface. In the case of CA = 105° and 55° of the interlaced surface, the CHF was enhanced by approximately 65% as compared with that of the plain surface. In another plain-hydrophilic interlaced case, the CHF of CA = 97° and 10° was approximately 840 kW/m^2^, and the CHF was enhanced by approximately 29% as compared with that of the plain surface. In other words, Figure 2 indicates that CHF values of the interlaced patterned surface were higher than those of the isotropic plain surface because the bubble motion on the interlaced wettability surface increased HTC during pool boiling heat transfer. These results indicate that the difference of CAs (Δ*θ*) of the interlaced surface may affect the CHF of pool boiling heat transfer. The Δ*θ* of the interlaced surface affects the difference of CHF values (ΔCHF), as illustrated in Figure 5. The Δ*θ* is defined as CA of the plain region minus CA of the coated region. ΔCHF is defined as CHF of interlaced wettability minus CHF of isotropic wettability. The results in the right side of Figure 5 show the ΔCHF of plain-hydrophilic interlaced surface (Δ*θ* is positive), and results of the left side show the ΔCHF of plain-hydrophobic interlaced surface (Δ*θ* is negative). The absolute values of Δ*θ* were higher, and ΔCHF values were lower. This observed influence can be explained by formed bubbles in the high surface energy region. In the case of CA = 97° and 10°, the bubbles start nucleating on the plain region (CA = 97°); however, the formed bubbles in the hydrophilic region (CA = 10°) were smaller than those in CA = 55°as shown in Figure 3. The smaller bubbles moved the plain region and gathered together to form a larger bubble. Subsequently, this larger bubble remained on the heating surface for a considerably longer period of time in the case of CA = 97° and 10°. The bubbles stagnated in the plain region and resisted a conduction heat transfer during the nucleate boiling. Contrarily, in the case of CA = 97° and 10° (Δ*θ* = 87°), the surface energy of the plain region is relatively lower than that of the hydrophilic region compared with the case of CA = 105° and 55° (Δ*θ* = 50°). Because of relatively lower surface energy, the nucleate bubbles stay longer on the plain region in the case of 10° and 97° and bubbles are larger in this case. This might also explain why larger bubbles remained on the heating surface for a considerably longer period. Third, in the interlaced wettability surface, the best enhancement of CHF might have existed in smaller Δ*θ*. Regarding bubble motion effect in the case of absolute values of larger Δ*θ*, the small bubbles must remain for a longer period of time to form a larger bubble and must depart later. In other words, higher ΔCHF are obtained when the absolute values of Δ*θ* are smaller. As mentioned, the hydrophilic region exhibited higher CHF values. In this study, hydrophilic and near-superhydrophilic interlaced surfaces can be fabricated, the CA values were 56° and 16°, and Δ*θ* was 40°. This case has good enhancement, the CHF increased to values of up to 1,420 kW/m^2^ and up to 100% of the values of the plain surface, as shown in Figure 4.

## Conclusion

In conclusion, this study has demonstrated that surfaces with interlaced wettability significantly enhance the CHF and the HTC due to the bubble motion during pool boiling. Interlaced surface of Δ*θ* is an important index to affect the pool boiling. A good enhancement arises in the case of hydrophilic and near-superhydrophilic regions, in which Δ*θ* is 40° in the hydrophilic region. The best enhancement of CHF might have existed in smaller Δ*θ*.

## Competing interests

The authors declare that they have no competing interests.

## Authors' contributions

PHC provided the idea and did the proofreading of the manuscript. CCH drafted and revised the manuscript. CCH and TWS designed and carried out the experiment. Both authors read and approved the final manuscript.

## Author's information

Prof. PHC received his master's and Ph.D. degree in mechanical engineering from the University of Minnesota in 1984 and 1988, respectively. He was awarded as a distinguished professor by the National Taiwan University in 2008, the distinguished research award by NSC in 2008 and 2011, and ASME fellow in 2009. His major research areas are in MEMS, biomedical devices, nanotechnology, and energy-harvesting chips, highly efficient energy systems, and sensors. He has published more than 127 journal papers, 2 books, and 14 patents.
